# Co-creating the Patient Partner Guide by a Multiple Chronic Conditions Team of Patients, Clinicians, and Researchers: Observational Report

**DOI:** 10.1007/s11606-021-07308-0

**Published:** 2022-03-29

**Authors:** Constance van Eeghen, Juvena R. Hitt, Douglas J. Pomeroy, Paula Reynolds, Gail L. Rose, Jennifer O’Rourke Lavoie

**Affiliations:** University of Vermont, Given Courtyard Fourth Floor S456, 89 Beaumont Avenue, Burlington, VT 05405 USA

**Keywords:** Stakeholder participation, Patient engagement, Multiple chronic conditions, Behavioral health integration, Research team development

## Abstract

**Background:**

Engaging patients as partners can influence research, with rewards and deterrents. The authors are researchers and patient co-investigators who collaborated on a comparative effectiveness, randomized controlled study of a structured quality improvement (QI) process to improve behavioral health and primary care integration for people managing multiple chronic conditions (MCC). Patient co-investigators responded to a gap in available resources to support study clinics in partnering with their own patients in QI and co-created the Patient Partner Guide (PPG).

**Objective:**

Describe the development of the PPG, its use by clinics undertaking the QI project, and research team partnerships.

**Design:**

Observational report of study intervention component.

**Participants:**

Diverse patients and family members managing MCC and members of their primary care clinics.

**Intervention:**

The PPG component of the study intervention is a five-step workbook providing practical tools and resources to sustain partnerships across clinic QI team members, including patient partners. The process of developing the PPG relied on relationship-building tools that were iteratively assessed, practiced, improved, and incorporated into the PPG under the leadership of patient co-investigators.

**Main Measures:**

Observations related to PPG use and patient partner inclusion in clinic QI; impact on the research team.

**Key Results:**

Of 20 clinics, 6 engaged patients as full partners on QI teams. Clinics found resistance in partnering and challenges in using the PPG but valued the material and their partners’ contributions. Similarly, engagement of patient co-investigators in research brought a shift in perspective to team members. The PPG is available and was adapted for use by research teams.

**Conclusions:**

Engagement of patients and other stakeholders in research can be transformative and productive. Building relationships through meaningful work benefits others, and in turn, the research process. This approach can enhance clinical care QI and may result in substantial contributions to the conduct of research.

**Clinical Trial Registration:**

https://clinicaltrials.gov/ct2/show/NCT02868983

**Supplementary Information:**

The online version contains supplementary material available at 10.1007/s11606-021-07308-0.

## INTRODUCTION

Engaging patients and stakeholders in research; a practice formalized in 2010 by the Patient-Centered Outcomes Research Institute (PCORI);^1^ offers multiple benefits such as greater relevance in research topics;^1, 2^ improved credibility of proposals and research instruments,^2^ increased patient enrollment; decreased attrition;^2, 3^ and faster approval of evidence by regulators.^2^ However, deterrents to engagement are real and may include increased time and costs of the research team,^2, 3^ tension related to poor clarity about roles,^1^ unmet expectations,^2^ lack of confidence,^2^ the risk of tokenism,^2, 3^ and the burden of responsibility.^2, 3^ Acquiring these benefits and managing the deterrents require a shared goal, a thoughtful structure, clear expectations,^3^ reciprocal relationships,^4^ and an open and trusting working atmosphere.^1, 5^ Achieving these prerequisites depends in part on how researchers engage stakeholders, particularly regarding the use of collaborative, rather than consultive, methods that correspond to greater patient influence.^6^

The PCORI Engagement Rubric^1^ offers principles for engaging patients throughout the research process from the generation of research questions to the dissemination of results. For example, patients in PCORI-funded research have assisted in developing patient-centered study websites and selecting patient-reported outcome measures.^7^ A key role for patient partners is identifying the topics, measures, procedures, outcomes, and communication strategies most relevant to them.^8^ Engagement may be influenced by patient partners’ motivations to participate in research: dissatisfaction with previous or current healthcare experiences,^9^ strong opinions on improvement for the benefit of others,^10^ beliefs in the value of the health intervention,^10^ or to fulfill their own desire to learn.^10^

The authors are collaborators on a large, pragmatic, comparative effectiveness randomized controlled trial designed to improve care and outcomes for people managing multiple chronic conditions (MCC). The study tested whether a structured quality improvement (QI) process undertaken at primary care practices seeking to integrate behavioral health and primary care (IBH-PC) would produce superior patient health and clinical outcomes when compared to practices undergoing no structured QI process.^11^ The research team included three patient co-investigators (co-authors JL, PR, and DP) from the inception of the study’s aims in 2015 through dissemination of its results in 2021. Each patient co-investigator had ongoing experiences with diverse presentations of MCCs and responded to invitations from providers to participate. The research team used a “study-focused” framework for involving patients in research,^12^ practicing thoughtful engagement processes tied to study activities to support collaborative work. Patient co-investigators, considered study key personnel, influenced study activities across three domains of engagement,^1^ including the following: (1) *planning the study*, by identifying meaningful patient-reported outcomes, inclusion criteria, and patient-centeredness themes and tools, and developing the Patient Partner Guide (PPG, described below); (2) *conducting the study*, by developing a common glossary of terms (Table 1 is an example for this report), contributing to the online learning community, and participating actively on the research team; and (3) *disseminating results*, by planning and delivering presentations, publications, and stakeholder convenings; creating peer-to-peer guidance for patients and clinical teams working together (Appendix: “Would you like to feel better?”); and publishing the PPG on the Internet.

**Table 1:** Glossary of Terms and Abbreviations

Behavioral health	Mental health, health behavior, and lifestyle that affect mood, stress, pain, sleep, medications, alcohol and substance use, weight, nutrition, and physical fitness
IBH-PC	Integrating Behavioral Health and Primary Care research study testing an intervention to create a collaborative system of care with behavioral health providers as full members of primary care health teams
MCC	Multiple chronic conditions, such as combinations of medical, mental, and behavioral health diagnoses
PCORI	Patient-Centered Outcomes Research Institute
PROMIS-29	29 items of the Patient-Reported Outcomes Measurement Information System, a survey completed by patients
PPG	Patient Partner Guide, a structured method for engaging patients in primary care quality improvement
QI	Quality improvement process for improving health care delivery
TIDieR	Format for reporting research interventions: Template for Intervention Description and Replication

This observational report highlights one of many productive patient partner contributions to the study: the development and use of the PPG as part of the intervention. Early in the design of the structured QI process, patient co-investigators identified the need for clinics to include their own patients in QI activities, echoing published recommendations.^13–20^ The goal of the PPG was to help clinics gain essential, patient-based insight to achieve patient-centered integrated BH services for patients managing MCC. Our patient co-investigators led the design of the PPG, bringing together diverse research team members. This report uses the Template for Intervention Description and Replication (TIDieR)^21^ format to describe the development of the PPG and its use by clinics undertaking the QI project.

## METHOD

### PPG Development

Following the PCORI engagement rubric,^1^ also included in the PPG, co-investigators started by creating an Engagement Team, co-led by a patient partner (JL) and project director (CvE). The Engagement Team included additional patient co-investigators (PR, DP) and worked with stakeholders with personal or professional MCC experiences to learn about engagement. This team drew from demonstrations of healthcare organizations that engaged stakeholders in advisory groups, shared governance, and internal quality improvement teams.^20, 22–24^ The Engagement Team was guided by sources that emphasized shared leadership as the peak of a graduated scale of partnerships built on a foundation of co-learning, trust, transparency, and honesty.^14, 25–27^

Engagement Team members assessed, practiced, and improved on methods to engage as multi-stakeholder partners, creating and using an approach to identify common values and goals. Patient co-investigators and stakeholders provided guidance on what matters most when including diverse stakeholders with MCC in QI efforts. Throughout the PPG’s development, team members created affinity diagrams,^28^ shared lived experiences, and used an appreciative response process to receive and provide feedback (see Appendix: “Offering Feedback”). These processes became part of the PPG to create a healthy, equitable, inclusive, and cooperative environment for all members of clinic QI teams.

In developing the PPG outline, the Engagement Team benefited from existing resources from the Robert Wood Johnson Foundation initiative, “Aligning Forces for Quality,” and others that explore patient involvement^18, 29–35^ as well as personal conversations with their authors. The Engagement Team heard the value of patience and persistence from these sources. From the study’s Stakeholder Advisory Group (SAG) members with experiences in MCC, the team learned the importance of positivity, appreciation, kindness, compassion, and, above all, the high value in the patient’s lived experience—the expertise of the patient. Engagement Team members revised each step of the PPG in cycles, engaging stakeholders in writing, review, and revising again. In its final presentation, the PPG speaks to two audiences: (1) clinic leadership and clinicians, to support the benefits and challenges of patient engagement in QI and (2) clinic patients and family members, to be welcomed and oriented to a QI team in simple, appreciative language.

### PPG Content

**Figure 1:**
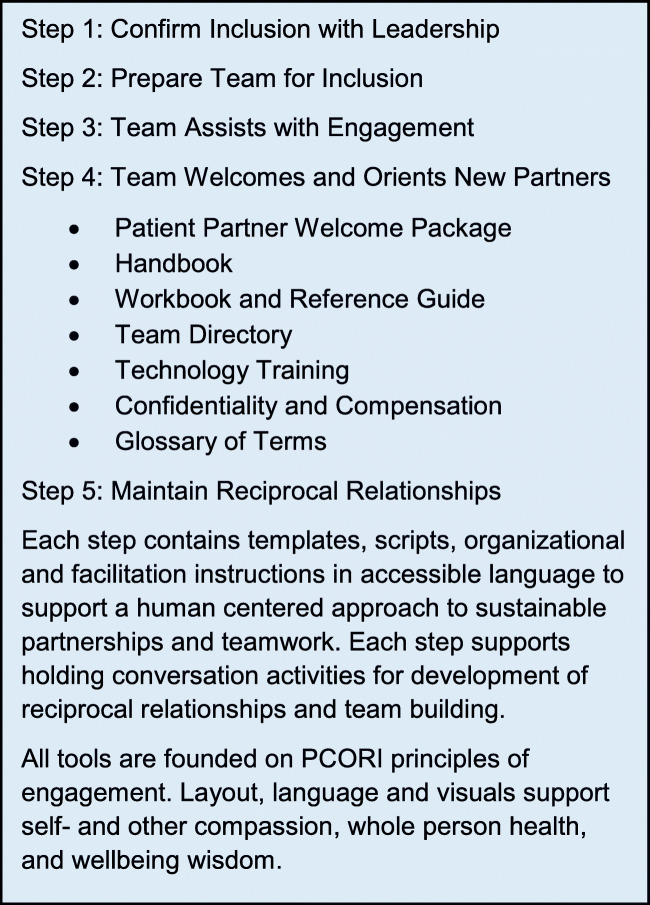
Overview of the patient partner guide.

The PPG, one component of the IBH-PC intervention, is a printable online document structured as a workbook. It includes five steps that can be followed sequentially or in the order most relevant to the practice (Fig. 1). PPG materials were specifically intended for use with patients who are managing MCC, providing a QI project Welcome Package and Overview to focus on improving care for patients who experience chronic, complex, and multiple health conditions. The PPG includes testimonials, practical guidance, patient-centered scripts and messaging, templates for contracts, spreadsheets to document progress, handbooks, instructions on compensation and confidentiality, and images to enhance the learning experience while sustaining partnerships with all team members. The PPG is framed by simple mnemonics (see Appendix: “The 6Ss” and “6Rs of a Clinical Team”) for successful teamwork and to reinforce the reciprocal relationships needed for equitable partnerships.

### PPG Delivery

The PPG was located on a private website available to QI teams to download step by step as needed while forming team relationships and following the QI project. QI teams included the role of “patient partner liaison,” a member from the clinic to assist in outreach and support, for example, by flexibly scheduling QI team time to include patient partners.

The PPG encouraged QI teams to adapt the materials to their local environment. In fact, templates, scripts, and handbooks were specifically created for customization, such as organizational rules regarding training in confidentiality and privacy. As recommended by the PCORI Engagement Rubric,^1^ the PPG included guidance on the value of and methods for compensating patient partners in contributing to clinic QI.

### Data Collection and Analysis

During the study’s active phase, remote coaches working with each QI team collected feedback on the PPG from team members, including their initial intention to include patient partners, modifications to patient partner inclusion as the project progressed, the timing of patient involvement related to when partnerships began, and open comments about the PPG. Data were recorded in a shared log by coaches, and reviewed, discussed, and verified at bi-weekly meetings with the lead patient co-investigator (JL). Coaches and patient co-investigators reviewed PPG data again after the end of the observation period to ensure data completeness and accuracy.

Categorical responses were analyzed with descriptive statistics, such as number of sites including patient partners and number of inclusion methods used. Coaches and patient partners, as an analytical team, reviewed and categorized qualitative feedback about the use of the PPG, identifying themes in both appreciative comments and suggestions for improvements. Themes were updated as new data emerged, grounded with direct quotations and re-evaluated to ensure that the meaning of the comment was accurately captured from the perspective of all team members. All categorical and qualitative results were considered in revising the PPG in repeated cycles of improvement.

The IBH-PC study was reviewed and approved by the University of Vermont Research Protections Office Institutional Review Board’s Committee on Human Research (CHRMS#16-554).

## RESULTS

### PPG Use and Feedback

**Figure 2:**
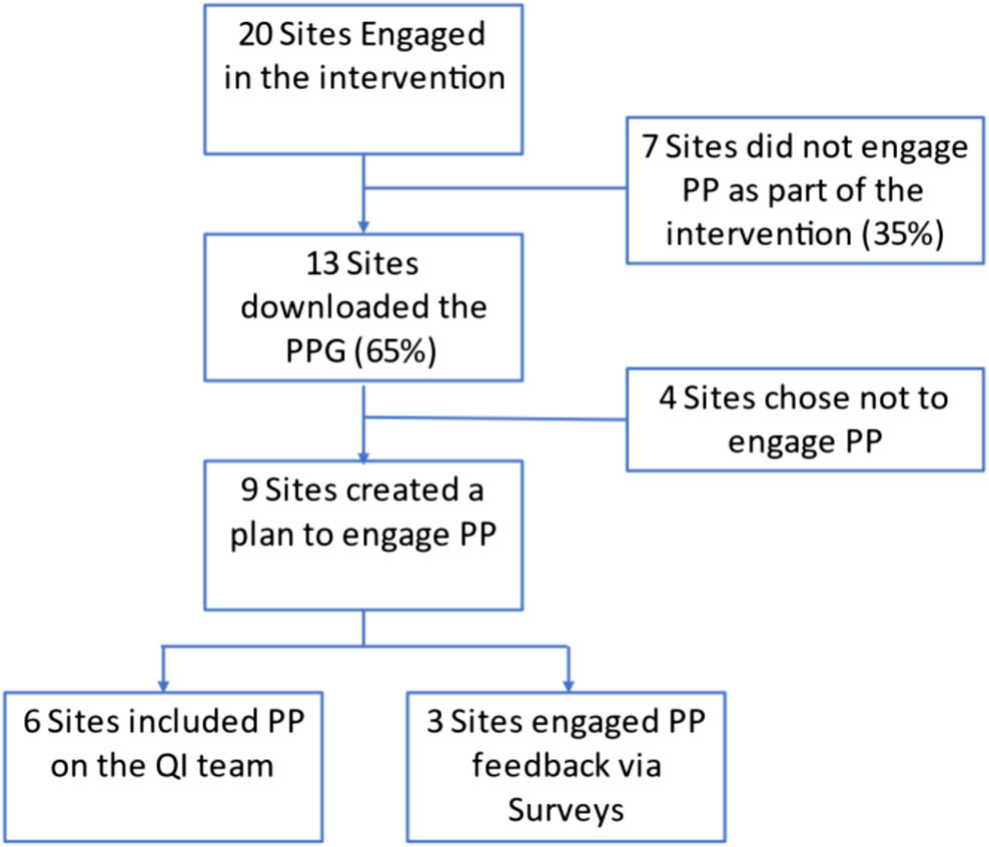
Use of the patient partner guide by clinic QI teams.

The active arm of the IBH-PC study for which the PPG was developed included 20 diverse primary care clinics across the USA described in detail elsewhere.^11^ Of the 20 clinics, 13 elected to download the PPG during the project period and nine created a plan to engage patients in QI. As guided by the PPG, six clinics engaged patient partners on their QI teams and three modified this direct engagement approach by collecting patient input via surveys. Of the six, two teams began their patient engagement at the start of their QI project, one in the middle of the project, and the remaining three engaged patients toward the end of their projects, as they tested new workflows in their clinics (Fig. 2).

Feedback from QI team members highlighted that inclusion of a patient partner on a QI team was simultaneously novel, beneficial, and initially daunting. Clinics noted that finding a patient partner with MCC required discussion among many clinic members, some of whom resisted the idea or expressed doubts about whether a patient would commit to regular meeting attendance. Although some teams worked diligently to recruit two patient partners, per the PPG, all six clinics engaged a single patient on a long-term basis. Typically, the patient who joined the team was someone well known to the clinic, who had previously volunteered, or who served as an advisor to the clinic or health system.

QI team members commented on the length of the PPG, technical challenges in downloading from a private website, and the positive value of the materials. Templates, for example, provided useful starting points for contracts with patients joining a QI team. In all six sites, patients operated as full participants in the QI teams. They attended regular meetings and, in four clinics, were paid for their efforts. They shared their own and family members’ experiences as patients at the clinic, asked clarifying questions, and expressed opinions about decisions the teams faced. Across all six clinics, QI patient partner input and feedback were valued, with one team reporting: “We changed our plan because of our Patient Partner.”

#### Impact on the Engagement Team

The effect of collaborating on this aspect of the research study as patient co-investigators and academic researchers was new learning for the authors, especially as the act of engaging patients as partners required a shift in understanding that affected everyone. In the words of one of the patient co-investigators (PR),

If you want transformation then you have to transform. Your organization, your process and your thinking must transform. The traditions and beliefs you may have will only be barriers if they are not allowing the patient to the table and to the meaningful transformation work that must be done.

Collaboration continues among research team members and patient co-investigators. For example, co-leaders of the Engagement Team and other stakeholders received an Engagement Award from PCORI to adapt the PPG for use by researchers to engage patients and other stakeholders in research, supporting mutually respectful relationships and processes among team members from varied backgrounds and perspectives (see Appendix for the link to the Partnering for Research website; PCORI EATR-18361).

## DISCUSSION

This report described the PPG and its development as part of a structured QI project in a study to improve the care and health of patients managing MCC. Patient co-investigators led the design of the PPG to help clinics engage their own patients as partners in QI. Almost a third of the QI teams included patients as full team members and reported value from patient partnering. Comments from QI team members indicated the PPG was influential in their decision and approach to engaging in a partnership with a patient.

Patient co-investigator leadership in and development of the PPG is a unique example of partner engagement in research. The PPG was conceived by patient co-investigators who saw an unmet need that they were ready to learn how to fill. They wrote, assembled, and curated the contents collaboratively based on stakeholder input, with suggestions from research team members.

Throughout the development and use of the PPG, research team members continued to learn about the importance of meaningful engagement in partnering^36^ and careful listening to the needs of patients and other stakeholders in their engagement as research partners.^37^ The bi-directional learning that comes from realizing that each team member has something important to offer the others was a satisfying aspect of this partnering.^38^ Work on the PPG depended on building reciprocal relationships and supporting co-production of meaningful outcomes.^4^ The intangible outcomes of this work have included greater equity, trust, and recognition of each other’s capacity to contribute value. These relationships have helped IBH-PC team members grow and develop as partners in health improvement and research.

Our experiences included some of the barriers identified in other studies.^3^ For example, the time needed to achieve a level of competency with communications technology was greater than expected. Stigmatizing medical terminology was an ongoing source of dismay for patient co-investigators. Also, time constraints limited researchers’ capacities to engage with patients as much as desired for full stakeholder participation. This was the first researcher-patient partnership for this team, and over time, sensitivity to dynamics needing attention, such as symptoms of disengagement or imbalances in power, increased. Regular check-ins and needs assessments helped prevent and address these symptoms and have informed the PPG. Taking time at the start or close of meetings to acknowledge each person was essential to the development of reciprocal relationships. The team depended upon individuals having the confidence to bring their best whole person health to the work. Checking in with each team member regularly became a norm for the Engagement Team. Additionally, patient co-investigators assigned to represent the identity of the MCC population discovered they needed to work doubly hard to maintain their healthy self-image and address their life/work balance throughout the study.

In addition to the team development needed to support a research study, engagement is an iterative process that begins before and continues over the course of and after the study. This process is an important part of team maintenance.^39^ Research constantly builds on past discoveries and identifies new opportunities that appear on the horizon. To act on those opportunities nimbly, stakeholder relationships are best if continually nourished. Periodic updates, whether through correspondence or virtual team visits, to evaluate future research opportunities, complete surveys on topics of interest, or share questions with peers are all part of nurturing the team and preparing the community for future work. One-on-one crucial conversations,^40^ if practiced regularly and used when needed, can resolve conflict and enhance team members’ commitment to project goals and support of one another through challenges. Engaging new partners in each of these opportunities builds a sustainable research stakeholder community.

This observational report has several limitations. Those QI teams that chose to use the PPG were self-selected and represent a small group of primary care practices. Results from this study are not generalizable to other practice teams. Also, the results reported here are limited to descriptions of the benefits and challenges of PPG use and do not include results of the QI teams’ work or its impact on patient health.

This study observed whether patient partners could engage in a research project by adding to a part of the intervention, thereby bringing patient engagement into primary care practices. Future research examining the impact of such engagement may strengthen commitment to such diverse partnerships. Future studies that engage patient and other stakeholder partners will likely need to assess their access to synchronous and asynchronous Internet platforms. Technology is a prerequisite to engagement for partners whose management of their MCC prevents travel for on-site meetings. While orienting patients to technology was a substantial hurdle in 2016, the near-universal transformation in communication practices resulting from COVID-19 dramatically shrank the technology gap for engaging new partners. Although facility with communication technology in the general population has grown, it cannot be assumed for all partner populations.

In addition, attention is needed regarding the degree to which partners in research are treated equitably by system structures, such as payments (salaried vs hourly employees or as unpaid volunteers). All authors agree that the communication methods used and compensation provided in the IBH-PC study were supportive of commitment, a culture of sharing, and the practice of dialogue that created the PPG. Understanding and improving key processes of patient engagement in research will continue to lead to new opportunities to standardize research practice. For example, developing a standard process to engage patient partners in examining a proposed intervention and its interface with study participants is a potential step that can build on comparative effectiveness research standards, as proposed by Esmail et al.^41^

The PPG is freely available as part of the study intervention to help QI teams in primary care clinics engage diverse stakeholders at https://sites.google.com/view/ibhpc/workbooks/patient-partnering.

## CONCLUSIONS

Engagement of patients and other stakeholders in research is dependent on a deliberate process to support reciprocal relationships in meaningful work. This example of a multi-stakeholder research team’s development of an intervention component to engage clinic patients in QI demonstrates that patients can do more than contribute to research. They can take leadership in identifying gaps in research concepts and tools and in responding to them, thereby making engagement successful and rewarding for all team partners involved.

## Supplementary information

ESM 1 (DOCX 3491 kb)
