# Supplementary material for: Co-creating the Patient Partner Guide by a Multiple Chronic Conditions Team of Patients, Clinicians, and Researchers: Observational Report
Source: J Gen Intern Med. 2022 Mar 29;37(Suppl 1):73–9. doi: 10.1007/s11606-021-07308-0 (PMC8960693; doi:10.1007/s11606-021-07308-0)
Supplement: Supplementary file 1 — (DOCX 3491 kb) [file 11606_2021_7308_MOESM1_ESM.docx]

**Appendix**

| Patient Partner Guide Materials Overview | 2 |
| --- | --- |
| PPG Sample: Offering Feedback | 3 |
| PPG Sample: The Basics for Effective Participation – The 6Ss | 4 |
| PPG Sample: Understanding the 6Rs of a Clinical Team | 5 |
| Patient to Patient: Would You Like to Feel Better? Control of Your Health | 8 |
| Partnering for Research website and PCORI engagement resource | 10 |

Patient Partner Guide Materials

List of Contents

<https://sites.google.com/view/ibhpc/workbooks/patient-partnering>

**Patient Partner Liaison Leadership Tasks:** materials to guide the quality improvement team in championing and leading patient partner engagement

1. Reflect Before You Begin
2. Including Patients as Partners
3. Confidentiality Measures and Agreements
4. Compensating Patient Partners Fairly
5. The Patient Partner Liaison
6. Troubleshooting
7. Patient Centered Rules of Engagement

**Patient Partner Welcome Package**: materials to shape the messages, instructions and expectations of orienting a new patient partner to the clinic’s quality improvement team

1. PP Welcome Letter
2. PP Checklist
3. Overview of the Initiative and Educational Program
4. Terms of Our Agreement
5. Patient Partner Contact Information
6. Patient Partner Sample Invoice
7. Patient Partner Name
8. Patient Partner Sample Honorarium Letter
9. Patient Centered Rules of Engagement
10. Patient Partner Handbook
11. Workbook and Reference Guide
12. Glossary of Terms

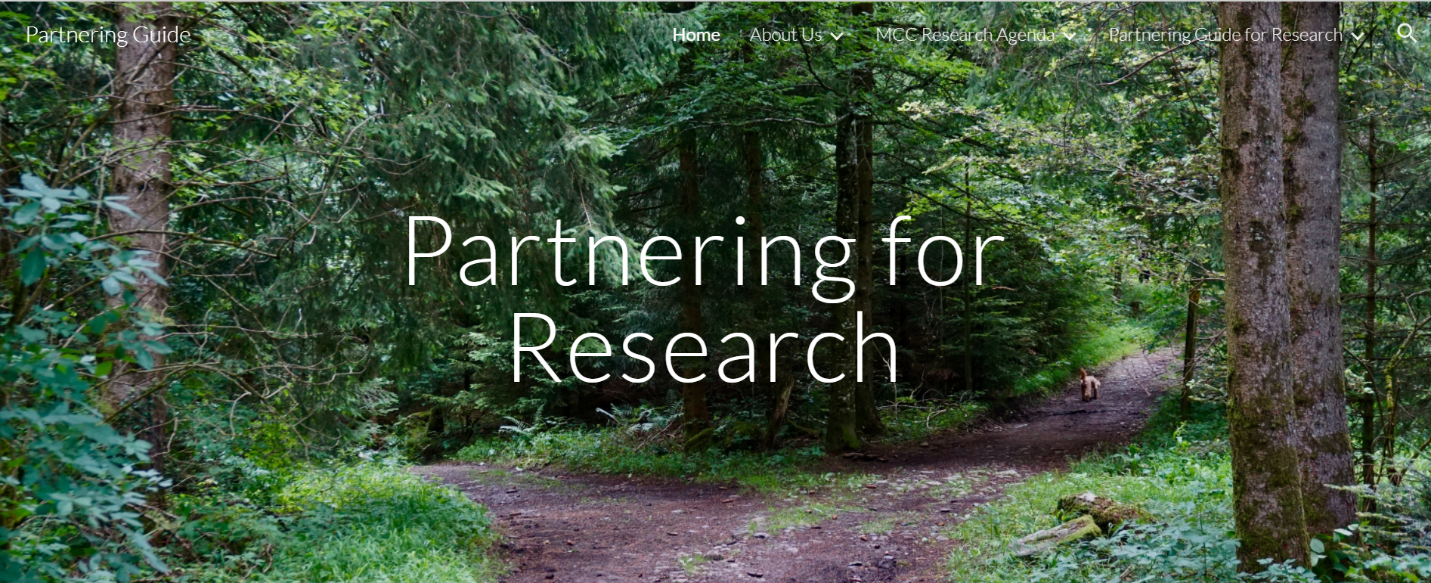


The Partnering for Research website, adapted from the original IBH-PC Patient Partner guide contains methodology as well as tools used to develop a new guide, a multiple chronic conditions research agenda and a roadmap for engagement. To explore the website, please visit:

<https://sites.google.com/view/partneringguide/home>

For more information on the PCORI engagement award which funded the multi-stakeholder community project called *Cooperatively Inspired Research Community for Learning and Engagement* (CIRCLE), during which the Partnering for Research guide was adapted, please visit:

<https://www.pcori.org/research-results/2020/pcorcer-partnering-guide>
